# Scalable Manufacturing Process and Multifunctional Performance of Cotton Fibre-Reinforced Poly(Lactic Acid) (PLA) Bio-Composites Coated by Graphene Oxide

**DOI:** 10.3390/polym14193946

**Published:** 2022-09-21

**Authors:** Yilin He, Shuying Wu, Anthony Chun Yin Yuen, Feng Huang, Cyrille Boyer, Chun H. Wang, Jin Zhang

**Affiliations:** 1 School of Mechanical and Manufacturing Engineering, University of New South Wales, Sydney, NSW 2052, Australia; 2 School of Engineering, Macquarie University, Sydney, NSW 2109, Australia; 3 School of Chemical Engineering, University of New South Wales, Sydney, NSW 2052, Australia

**Keywords:** biocomposite, physical properties, microstructural analysis, compression moulding

## Abstract

Natural fibre biopolymer composites with both fibres and matrix being derived from biomaterials are increasingly used in demanding applications, such as sensing, packaging, building, and transport, and require good electrical, thermal, and flame retardant properties. Herein, an investigation of the effectiveness of functionalising nonwoven cotton/poly(lactic acid) (PLA) fibre mats with graphene oxide nanosheets has been reported by using a facile dip-coating method followed by thermal reduction for enhancing the electric, thermal, and abrasion-resistance properties. The manufacturing processes for preparing biocomposites and introducing functionality are readily scalable. Experimental results reveal that with the addition of less than 0.5 wt% graphene nanoplatelets, the biocomposites showed significant improvements in abrasion resistance, electrical conductivity, thermal conductivity, and diffusivity. Furthermore, the composite shows excellent piezo-resistivity to act as strain sensors with a gauge factor of 2.59 at strains up to 1%.

## 1. Introduction

Natural fibre biopolymer composites are increasingly used in civil engineering due to their low environmental impact, low cost, and low emission of toxic fumes [1,2,3,4] when subjected to heat. Fire safety is a critical requirement for buildings, vehicles, and other engineered structures whose failures in the event of fire will cause loss of life or major property damage. At 300–500 °C, most synthetic polymers decompose, and the decomposition releases heat and toxic volatiles together with combustible gases, non-combustible gases, solids (usually char), and entrained solid particles (smoke), which may cause hazards such as loss of physical integrity, melting, and dripping thereby providing other ignition sources [5,6]. Therefore, the adoption of more natural fibre biopolymer composites in building materials will have advantages over synthetic fibre-reinforced polymer matrix composites [7]. 

Moreover, high resistivity is the main cause of accumulation of static electricity and electrostatic discharge in polymer composites, leading to many explosion incidents in underground mines, damages to satellites and sophisticated electrical/electronic components and systems, and fire and casualties in oil–gas storage and transportation. This results in billion-dollar losses each year. To avoid these problems, either antistatic agents or conductive fillers are generally employed to reduce the surface or volume resistivity and to accelerate the dissipation of the high electrical charge on the surface of composites [8,9]. 

The introduction of conductive carbon nanofillers can also add multiple functions to composites that expand their use in numerous applications, including sensing for structural health monitoring, electromagnetic interference shielding, and thermal interface materials. Since graphene has a high surface area, aspect ratio, tensile strength, thermal and electrical conductivities, and low thermal expansion coefficient, and can be conveniently derived from graphite in large quantities, it is widely used to enhance mechanical, electrical, and thermal properties of composites [10,11,12]. Moreover, graphene has been shown to be a promising component for innovative strain sensors. For instance, laser-scribed graphene foam [13,14,15], graphene aerogel [16], and suspended graphene membranes [17,18] have demonstrated high piezo-resistivity, making them emerging solutions for new strain sensors. Grafting graphene oxide (GO) onto the carbon fibre surface has been shown to effectively improve the interfacial property of carbon fibre composites, e.g., an improvement of 55% in interlaminar shear strength was found, which confirms the enhancement of interfacial adhesion [19,20]. Similarly, the GO coating on short or continuous glass fibres also enhances the interfacial adhesion that results in increased tensile and flexural moduli [21,22,23]. 

There has been rapid growth in research and innovation for endowing composite structures with multiple functionalities [24,25,26]. Plant fibres are the most frequently used natural fibres for bio-composite reinforcement; among them, cotton fibres have successfully reinforced geopolymer [27], thermoset resin [28], and thermoplastic resin [29]. Poly(lactic acid) (PLA), bioderived from starch, is popularly used as a biodegradable matrix ascribed to its good mechanical properties and has shown to exhibit higher strength with natural fibres than polypropylene (PP) [30,31,32]. Although the addition of carbon nanofillers to fibre-reinforced composites creates multiscale and multifunctional composites with enhanced mechanical properties and damaging sensing capabilities owing to the electrical resistance change [33], effectively introducing these nanofillers into the composites remains difficult. Because of the tendency to form agglomeration, it remains a great challenge to uniformly disperse nanofillers within the polymer matrix [34]. 

Graphene and its derivatives, GO and reduced graphene oxide, can be introduced into polymer matrices by dispersion to enhance mechanical, electrical, and other properties. For example, GO can be easily processed with water-soluble polymers. The addition of 0.3 wt% of GO nanosheets into a water-soluble polyvinyl alcohol (PVA) matrix has shown to increase the tensile failure strain from 72% to 237% by simple ultrasonic dispersion [11]. The inclusion of graphene nanoplatelets into the PLA matrix, however, requires energy-intensive extrusion processes for blending these carbon fillers into the melted PLA matrix [35]. Alternatively, graphene has been applied as a coating onto biopolymer surfaces instead of bulk additives for added functionality, such as the gas barrier property, which overcomes common issues related to the dispersion of graphene in the polymer matrix. This is, nevertheless, limited to the very thin surface layer, and the bilayer structure is prone to delamination or generation of microcracks due to the insufficient adhesion between the nanosheets and the polymer [36]. In this work, the advantages of GO water solubility (environmental friendliness), uniform fibre mixing by textile carding process with homogeneous fibre reinforcement arrangement, and the resulting graphene filler uniform distribution after dip coating and thermal reduction have been taken advantage of for the preparation of strongly bonded graphene coatings on biocomposites by the facile and scalable manufacturing approach in this work. 

This work has demonstrated a scalable, facile method to create highly conductive, abrasion-resistant coating on natural fibre composites and studied their piezo-resistivity for the application of strain sensors. The coating was achieved by dipping cotton/PLA (50:50) nonwoven fibre mats in a GO water solution. The coated fibre mats were then consolidated in the hot press at an elevated temperature while the GO was reduced simultaneously. By reducing graphene oxide, the oxidised functional groups were removed to obtain a graphene material called reduced graphene oxide, often abbreviated rGO [37]. As a result, bio-nanocomposites, i.e., cotton fibre-reinforced PLA matrix composites, were prepared with homogeneously distributed rGO nanofillers as coating. The simple dip coating approach followed by the simultaneous reduction during curing significantly reduces the process complication usually incurred in conductive composite production, which paves the way for large-scale industry manufacture. The effect of the filler content on the fibre and composite morphology, dc electrical conductivity, thermal properties, and nanoindentation behaviour of the composites has been reported. The electromechanical response was also investigated under both quasi-static monotonic and dynamic cyclic tensile loading tests.

## 2. Experimental

### 2.1. Synthesis of GO Nanosheets

GO nanosheets were prepared using a modified Hummers method [38]. Graphite flake (10 g) and sodium nitrate (7.5 g) were added to 300 mL of sulfuric acid (98%), and then 40 g of potassium permanganate was added to the reaction slowly over one hour. The mixture was stirred at room temperature for 3 days, followed by adding 1 L of hydrogen peroxide solution (1% in water). The mixture was then filtered and washed with deionised water until pH 7. The resulting black cake was re-dispersed in deionised water to give a dark brown dispersion, which was then subjected to dialysis for one week to remove the residual salts and acids. The brown suspension was dried at 40 °C under vacuum. The synthesis resulted in approximately 188 g of graphitic oxide containing 23% water and 2% ash. Therefore, the concentration of GO obtained was 75 wt%. GO suspension was then obtained by sonicating the as-prepared black solid in water under ambient conditions for 30 min.

### 2.2. Fabrication of Graphene Nanoplatelet (GNP)-Coated Cotton/PLA Composite Laminate

**Figure 1:**
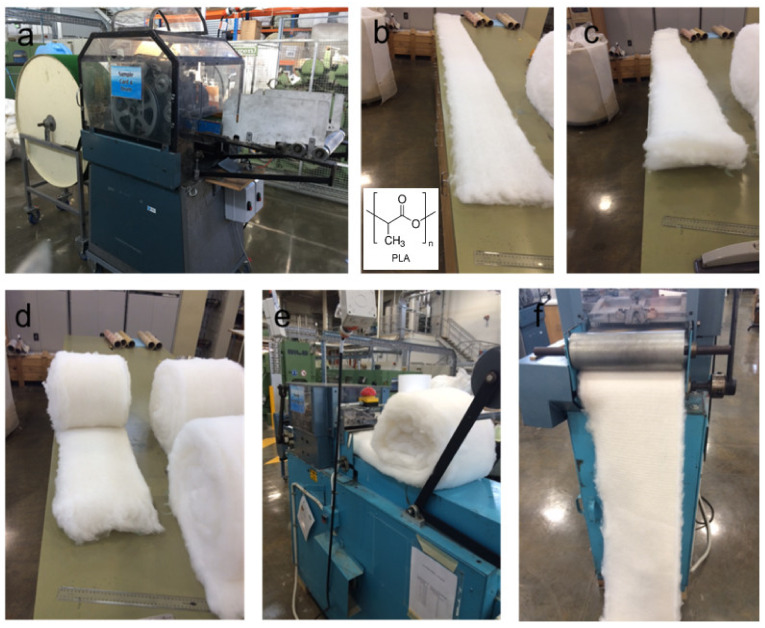
Processing nonwoven mats from cotton and PLA fibres. (**a**) Carding machine; (**b**) equivalent weight of cotton fibres and PLA fibres were hand blended firstly before putting through a carding machine twice for uniform mixing; (**c**,**d**) webs were then stacked into multiple layers; (**e**) 12 layers of webs went through needle punching machine at 30 punches/cm^2^ on both sides; (**f**) nonwoven mats for the manufacture of composite panels.

Cotton fibres (4.5 micronaire and 32 mm in upper half mean length) were supplied by Commonwealth Scientific and Industrial Research Organisation (CSIRO), Australia. The PLA fibres, with a fineness of 6.7 dtex, and a length of 64 mm, were supplied by Max Model under the trademark of Ingeo. The cotton fibres and PLA fibres were opened, blended, carded, laid up, and finally needle-punched, forming a homogeneous nonwoven mat. Prior to carding, both the cotton and PLA fibres were hand-opened to feed the carding machine (Figure 1). Webs of approximately 300 mm by 3000 mm were produced before subsequently being stacked into a total of 12 layers and needle punched on both sides at 30 punch/cm^2^ on a Fibre Locker (James Hunter, North Adams, Mass., USA). The area density of the cotton/PLA nonwoven mat was 1351 g/m^2^. For the first coating cycle, the nonwoven mat was dipped in the GO water suspension for 3 min and then removed. The GO dip-coated mat was then dried in air at room temperature for 24 h. Subsequent coatings were applied following the same procedure up to the 6th coating cycle. Finally, the dried coated nonwoven mats and the cotton/PLA mat without coating were dried at 60 °C for 24 h in the vacuum oven before being consolidated in a hot press at 185 °C for 15 min under a pressure of 1250 psi. The consolidated composite panels were then heat-treated at 120 °C for 30 min in an oven. The pristine cotton/PLA composite laminate (CP) and the coated laminates (CP_0.19GO, CP_0.38GO, and CP_0.51GO) were prepared for further characterisation. The GO concentration is 0.19 wt% for nonwoven mats with two coatings, 0.38 wt% for that with four coatings and 0.51 wt% for that with six coatings. The consolidated laminates have a thickness of 1.6~1.8 mm.

### 2.3. Characterisations

The morphologies and size of the prepared GO nanosheets were investigated by atomic force microscopy (AFM) on a Bruker MultiMode-8 tester. Fourier transform infrared (FTIR) spectra of GO and r-GO were measured on a Bruker Vetex-70 FTIR spectrometer and recorded in the range of 600–4000 cm^−1^ at a resolution of 4 cm^−1^. Nanoindentation tests were performed with an Ultra-Micro Indentation System II using a Berkovich indentor. The thermal properties of composites were measured by an LFA 457 Laser Flash Apparatus. The electrical resistivity of the composite laminates was measured using the SZT-2A Four-point probe tester, while a direct current range of 0~100 mA was chosen for the measurement. Three-point bending tests were also conducted to investigate the influence of GNP addition on the mechanical property of the modified composite laminates using an Instron 30 kN 5967 at a loading rate of 1.33 mm/min in accordance with the ASTM D7264 standard. The flexural specimens had dimensions of 50 mm × 12.7 mm, and the span length for the testing was 40 mm. 

The flexural stress was calculated for any point on the load-deflection curve by the following equation:

(1) $$\sigma =\frac{3PL}{4bh^{2}}$$

where *σ* is the stress at the outer surface in the load span region (MPa), P is the applied force, (N), L is the support span (mm), b is the width of the beam (mm), and h is the thickness of the beam (mm). The flexural modulus of elasticity was calculated as:

(2) $$E=\frac{L^{2}m}{4bh^{3}}$$

where *E* is the modulus of elasticity, m is the slope of the force-deflection curve. 

Optical images were taken using an Olympus DP71 and the SEM images were obtained from a Zeiss Supra 55VP FEG SEM. The piezoresistive response of the fabricated GNP-coated cotton/PLA composite laminates was characterised by measuring the variation of the DC electrical resistance as a function of the applied tensile strain under quasi-static monotonic and dynamic cyclic loading conditions. Specimens of 40 mm × 7 mm × (1.6~1.8) mm dimension were tested. The displacement-controlled static loading was carried out at a strain rate of 6%/min, while the cyclic loading was carried out by applying sinusoidal cyclic load at a frequency of 0.08 Hz. In situ change in resistance was measured by a two-probe method using a Keysight Technologies 34465A multimeter during mechanical tests.

## 3. Results and Discussion

### 3.1. Morphology of Graphene Oxide Nanosheets, Fibrous Nonwoven Mats and Cured Composite Laminates

**Figure 2:**
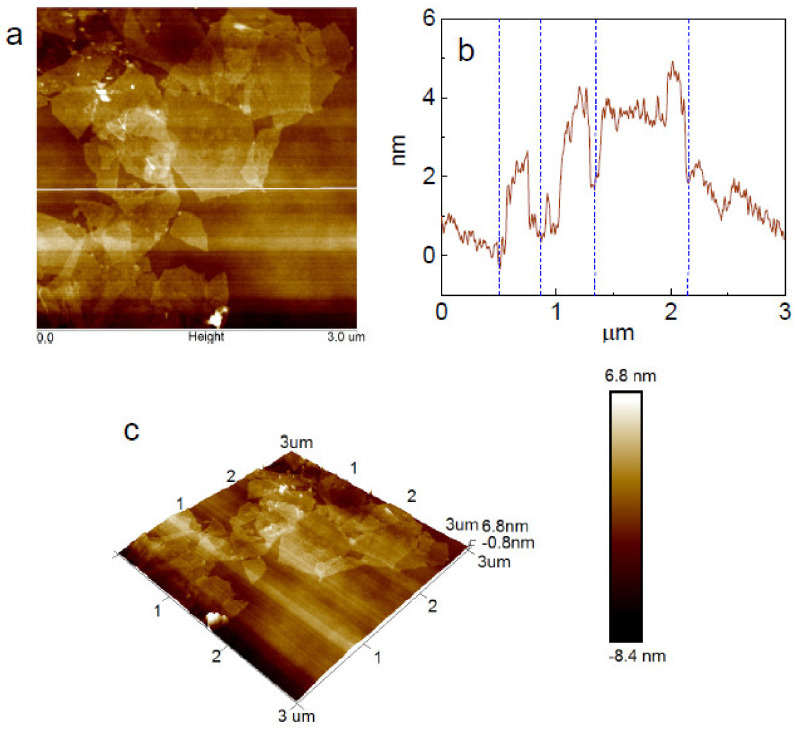
AFM images of the prepared graphene oxide nanosheets; (**a**) 2D height image; (**b**) section analysis; (**c**) 3D height image.

**Figure 3:**
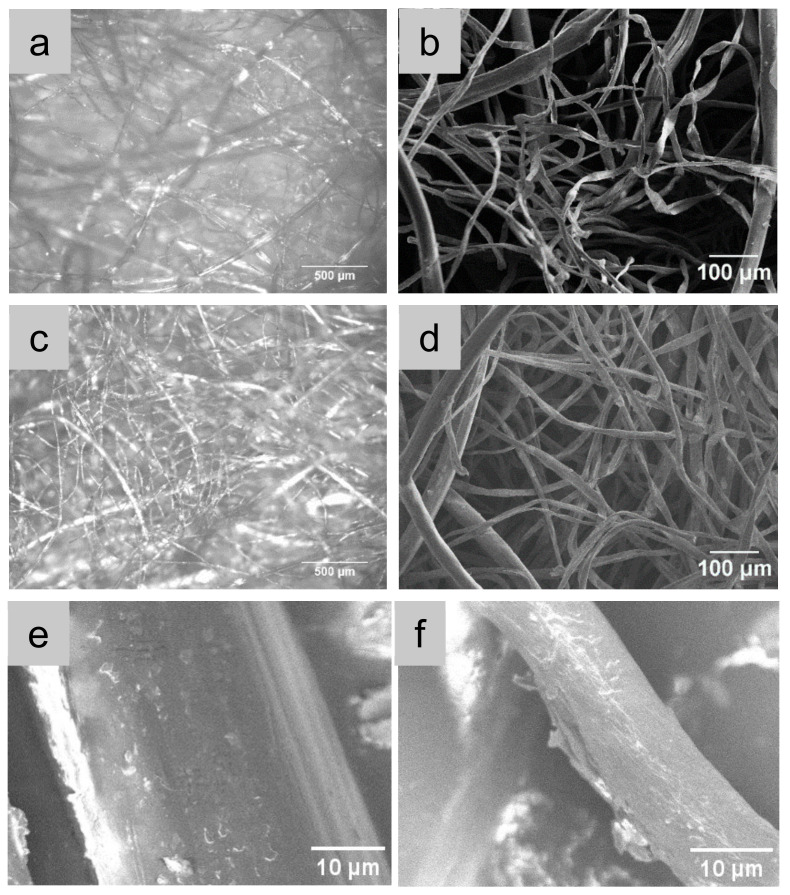
Morphologies of cotton/PLA nonwoven mat and the GO-coated cotton/PLA nonwoven mats. (**a**,**b**) Optical and SEM images of CP, respectively; (**c**,**d**) optical and SEM images of CP_0.38GO, respectively. (**e**,**f**) GO-coated PLA fibre and cotton fibre, respectively.

The synthesised GO nanosheets were characterised by AFM. The AFM images in Figure 2 show that the GO nanosheets were around 2 to 4 nm thick, and the lateral size of the nanosheets can be as large as 1 μm. As can be seen in Figure 3, the cotton and PLA fibres had different fineness. Since 4.5 micronaire converts to 1.77 dtex, the PLA fibres were around three times thicker than the cotton fibres. The finer fibres shown in the optical micrographs are cotton fibres, and the thicker ones are PLA fibres. The percentage weight increment of the nonwoven samples for successive coating cycles was approximately 0.19% for two coatings. The GO adsorption to the nonwoven fibre surface is influenced by the concentration of the GO suspension, the fibre surface tension and the surface area of the fibres [39]. The hydrophilic nature of the GO due to its functional groups, such as carboxyl, carbonyl, and hydroxyl groups, allows molecular level solubility in water that results in strong adhesion to the nonwovens [40]. After dip coating, there was no obvious variation in the optical morphology, although the colour of the nonwoven mats changed from white to brown; the high magnification SEM image reveals the presence of GO nanosheets on both PLA fibres (Figure 3e) and cotton fibres (Figure 3f). 

**Figure 4:**
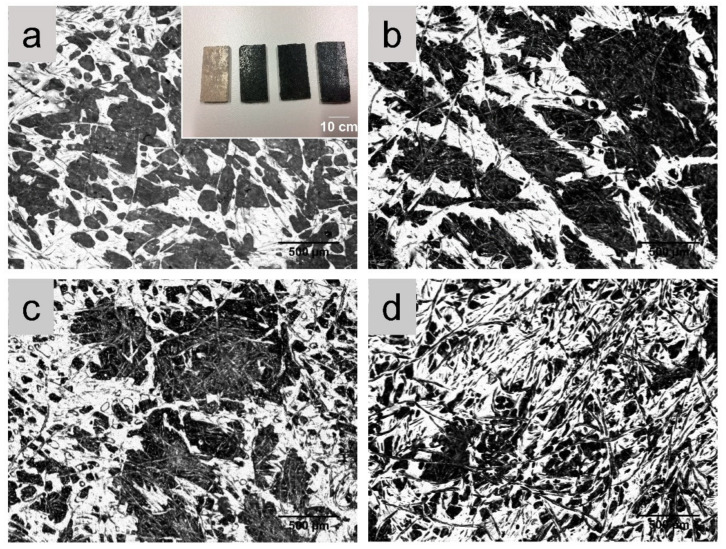
Optical images on the surfaces of the consolidated cotton/PLA composite and GNP-coated cotton/PLA composites. (**a**) CP; (**b**) CP_0.19GO; (**c**) CP_0.38GO; (**d**) CP_0.51GO. The inset in (**a**) is the photo of the four types of consolidated composite specimens sequentially.

After hot-pressing, the composite nonwoven mats transformed into laminates. The GNP-coated composite laminates were black due to the thermal reduction of GO during the hot-pressing of the composites. The higher the GNP loading, the darker the composite laminate (as shown in the inset image in Figure 4a). The 50:50 weight ratio between cotton and PLA led to the fibrous structure of the consolidated composite laminates, which is demonstrated in the optical images in Figure 4.

**Figure 5:**
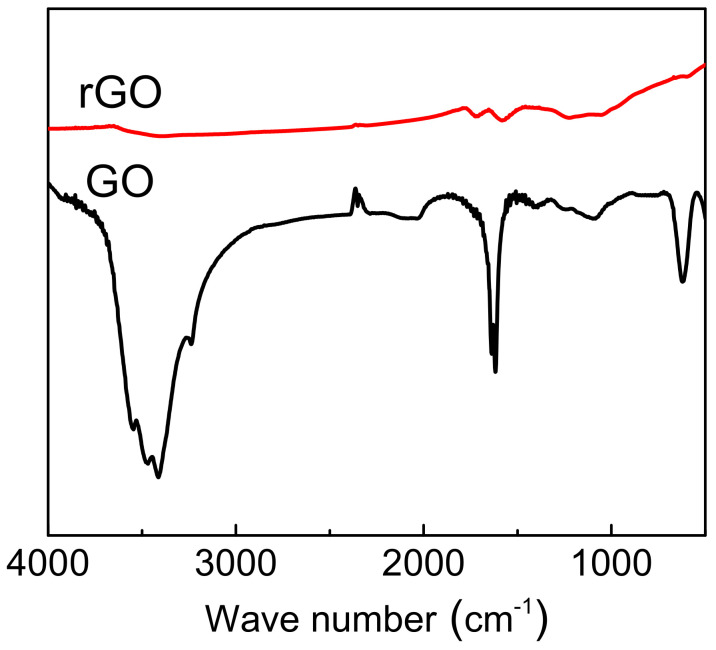
FTIR spectra of graphene oxide (GO) and reduced graphene oxide (rGO).

To confirm the thermal reduction of GO through the composite manufacture route, both the dried GO nanosheets and those thermally treated with the same temperature schedule as the composite panel fabrication were ground to KBr pellets for FTIR characterisation. It is shown in Figure 5 that the characteristic absorption bands of oxide groups in GO were the O-H stretching vibration in the broad range of 3200~3600 cm^−1^ [41], C=O stretching vibration peak at 1720 cm^−1^, HOH bending vibration at 1620 cm^−1^, vibration and deformation of O-H groups at 1471 cm^−1^, and the C-O (epoxy) stretching vibration at 1243 cm^−1^ [42,43]. These characteristic bands of GO decreased dramatically, and some of them disappeared entirely after the reduction treatment, indicating that most oxygen-containing functional groups in the GO were removed after the thermal treatment.

### 3.2. Nanoindentation Properties on the Surface of Composites

**Figure 6:**
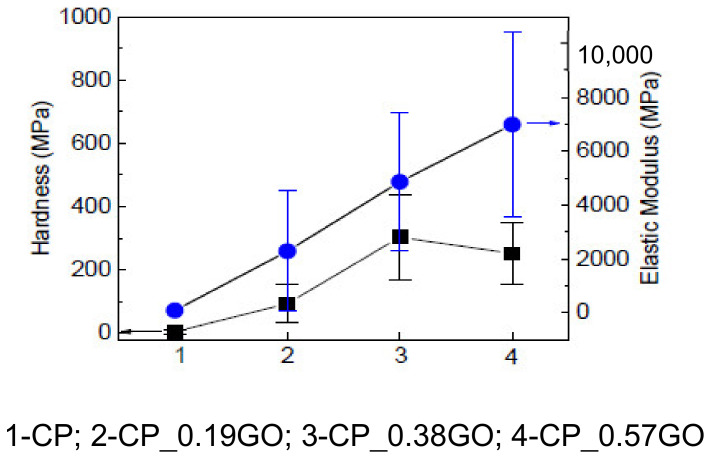
Nano-indentation properties of cotton/PLA composites with and without GNP coatings.

The introduction of GNPs significantly enhanced the hardness and elastic modulus of the surface of cotton/PLA composites (Figure 6). With the increase of GNP loading, the hardness increased from 3 MPa for the CP to 90 MPa for the CP_0.19GO, then changed to 300 MPa for the CP_0.38GO before a slight decrease to 250 MPa for the CP_0.51GO. Similarly, the elastic modulus improved from 66 MPa for the neat cotton/PLA composites to 2 GPa after the application of two coatings of GO, which is nearly a 33-fold enhancement for such a small percentage of GNP addition. With further increase in GNP loading, the elastic modulus continuously increased up to 7 GPa for the CP_0.51GO. The improved surface mechanical properties help to resist abrasion and friction incurred during the use life of cotton/PLA composites. Graphene is an atomically thick, 2D nanomaterial that possesses extraordinarily high Young’s modulus, tensile strength, aspect ratio, and surface area [44]. The enhancements in modulus, hardness, and stiffness of the polymer incorporated with graphene nanosheets are widely reported. These benefits mainly result from the enhanced interfacial adhesion that enables the transfer of the mechanical properties of graphene. The improvements are greatly dependent on the load-transfer efficiency [45]. The fabrication method used in this work enables the uniform dispersion of rGO on the surface of the cotton fibre PLA composite that efficiently transfers mechanical loading through the well-bonded interfaces.

### 3.3. Surface Electrical Resistivity and Three-Point Bending Properties of Composites

**Figure 7:**
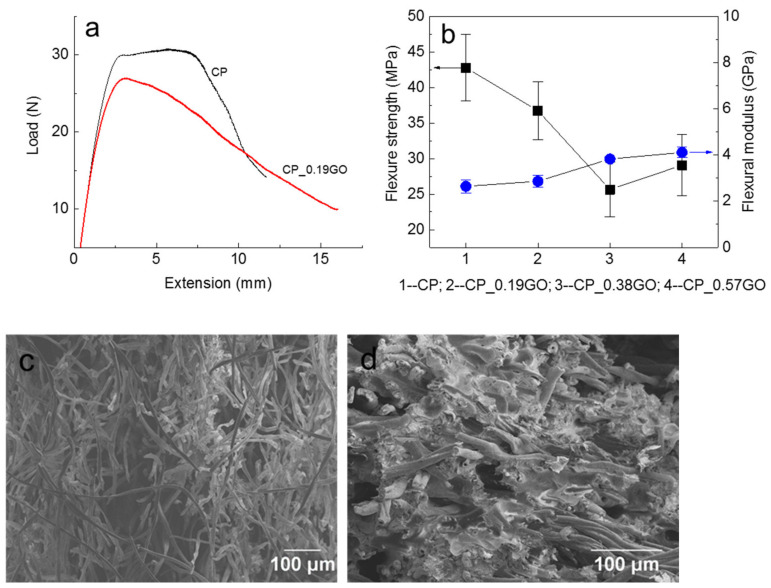
Three-point bending properties and fracture surface of the cotton/PLA composites. (**a**) The typical loading curves for the cotton/PLA composite and the GNP coated composite; (**b**) the flexural strength and modulus for each composite type; (**c**,**d**) flexural fracture surfaces of CP and CP_0.38GO, respectively.

With the increase in GNP content, the cotton/PLA composites transform from an insulator to conductive. The measured electrical resistivity was 6.10 Ω∙cm for the CP_0.38GO and 5.88 Ω∙cm for the CP_0.51GO, respectively. Since the addition of conductive nanofillers can reduce the strength of composites, the low percolation threshold facilitates the conductive surface without serious deterioration in mechanical properties [10]. These results are particularly useful for antistatic materials and electromagnetic interference shielding applications. The mechanical properties presented in Figure 7a,b show the decreased flexural strength and the increased elastic modulus for the composites as the content of the GNP increased. With the introduction of GNPs, the laminates became stiffer and less ductile. The adhesion between cotton fibres and PLA matrix became stronger with the addition of GNPs, as evidenced by the less fibrous fracture surface with blunt broken fibre cross-sections revealed by SEM (Figure 7c,d).

### 3.4. Thermal Behaviour of Composites

**Figure 8:**
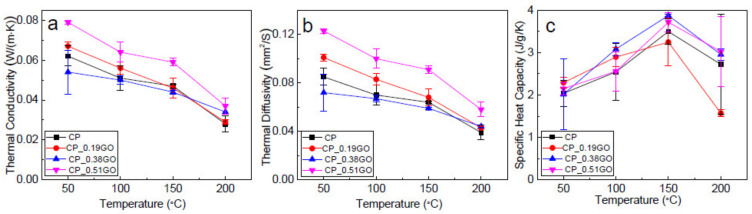
Thermal properties of cotton/PLA composites with and without GNP coatings. (**a**) Thermal conductivity; (**b**) thermal diffusivity; (**c**) specific heat capacity.

The thermal properties of the consolidated composites are presented in Figure 8. Thermal conductivity is the materials’ ability to conduct heat. From a mathematical perspective, it is described as a thermal energy Q transferred in a certain amount of time t, through the thickness x of the material and perpendicularly to the surface area A, due to a temperature difference ΔT between the opposite faces of the sample [46]. With the addition of such a small amount of GNPs on the surface of the cotton/PLA composites, the thermal conductivity of the composite laminate showed enhancement, as evidenced by the CP_0.51GO. The thermal conductivity of the CP_0.51GO was around 0.08 W/(m∙K), in comparison with 0.06 W/(m∙K) for the neat CP composite. Thermal diffusivity is the measure of thermal inertia. When a substance has high thermal diffusivity, heat moves through the substance rapidly because it conducts heat quickly relative to its volumetric heat capacity and does not require much energy transfer to or from its surroundings to reach thermal equilibrium [47]. Similarly, the thermal diffusivity increased as well due to the thermally reduced graphene oxide on the surface of the cotton/PLA composites. The mechanism attributed to the increase could be the presence of internal layers that enable phonon conduction and minimise coupling losses [48]. However, the heat capacity did not show obvious changes caused by the GNP coating. 

### 3.5. Electromechanical Response

**Figure 9:**
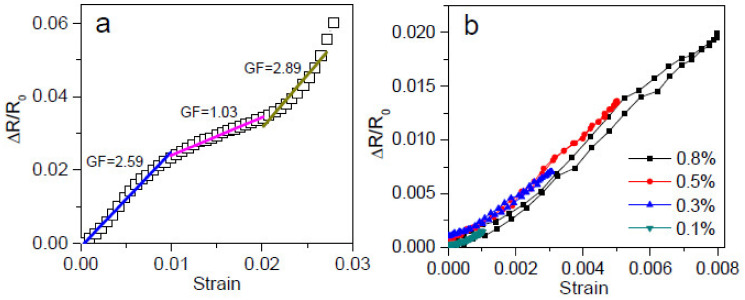
Relative change in resistance (Δ*R*/*R*_0_) with applied monotonic tension loading for the CP_0.38GO. (**a**) Up to failure; (**b**) under progressively increasing strain from 0.1%, 0.3%, 0.5% to 0.8%.

The piezoresistive behaviour of the GNP-coated cotton/PLA composites under static tensile loading was firstly analysed in Figure 9. Figure 9a presents the behaviour of CP_0.38GO and shows the relative change in resistance (Δ*R*/*R*_0_) with the strain level increasing up to failure. There is a quasi-linear relation between the relative change in resistance and the strain. Gauge factor (*GF*) was calculated using Equation (3).

(3) $$GF=\left(\Delta R/R_{0}\right)/\varepsilon$$

The gauge factor was 2.59 under 1% strain, 1.03 between 1% and 2%, and 2.89 between 2% and 3%, obtained by linear fitting of the (Δ*R*/*R*_0_) vs. *ε* curve. Similar to Wang et al.’s work, the different gauge factors at increasing strains were likely caused by the change in the overlapping area of the graphene nanosheets incorporated into the composites [49]. The resistance of the graphene composites was primarily dominated by the contact resistance of the sensing materials. With the extension of the graphene composites, the graphene nanosheets turned less compact, so the overlapping was reduced. The increased resistance can also be caused by the loss of contact between the adjacent nanofillers when subjected to stretching [50]. As a result, the resistive response exhibited in Figure 9a is influenced by the above factors in addition to the network structure of the melted PLA fibres reinforced by the cotton fibres deposited with GNPs. Due to the fabrication immersion conditions, the GOs were only able to get through the surface of the nonwoven mats at limited loading. The sensor demonstrates smaller sensitivity than the graphene sensor materials reported using viscoelastic polydimethylsiloxane (PDMS) as a matrix [51]. While the CP_0.38GO experiences stretching and releasing by tensile loading at a maximum strain level of less than 1% (Figure 9b), i.e., at the first stage of the Figure 9a curve, the relative change in resistance increases continuously upon stretching and then decreases gradually upon releasing with the GF equal to approximately 2.59. 

**Figure 10:**
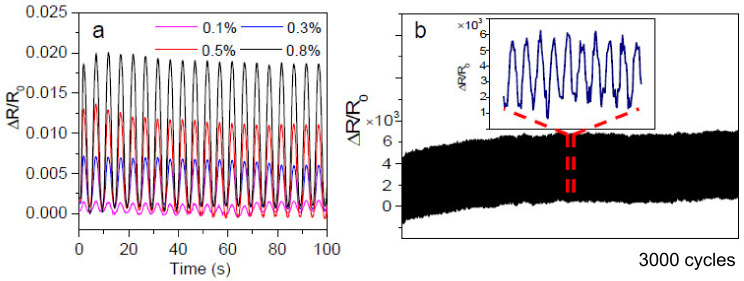
Relative change in resistance (Δ*R*/*R*_0_) with applied cyclic tensile loading for the CP_0.38GO. (**a**) Up to 10 cycles; (**b**) up to 3000 cycles.

The GNP-coated composites were also subjected to cyclic loading and unloading. Figure 10a presents the relative resistance changes of the composites being stretched to 0.1, 0.3, 0.5, and 0.8% and then released to 0% for ten cycles. For all the cycles, the composites recovered resistance after releasing the load, which indicates excellent durability. The nanocomposites were also subjected to cyclic loading and unloading up to 3000 cycles, and Figure 10b shows the resistance change. A continuous upward drift of the relative resistance change (Δ*R*/*R*_0_) was observed up to around 1000 cycles. The Δ*R*/*R*_0_ tends to stabilise thereafter, which demonstrates great durability. By dip coating the cotton/PLA nonwoven with GO and heat treatment afterwards, a natural fibre bio-composite sensor material was created with a gauge factor of 2.59. It has potential applications in structural health monitoring or other sensing purposes for lightweight automotive composite structures or lightweight sports instruments. These environmentally friendly bio-nanocomposites fabricated by a facile process demand proper mechanical conditioning procedures to minimise the effects of drift and hysteresis for improving repeatability performance and calibration procedures to compensate for the nonlinear sensor characteristic when strain is larger than 1%. 

**Figure 11:**
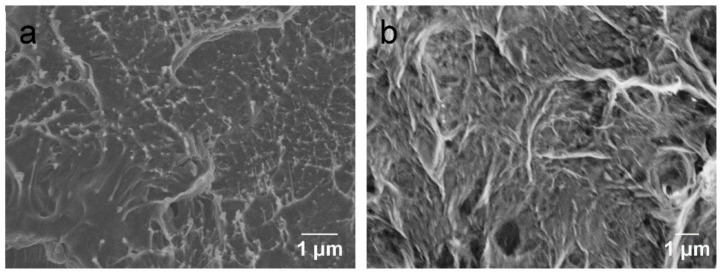
Comparison of fractography of consolidated nanocomposites (**a**) before and (**b**) after cyclic loading for 3000 cycles.

The SEM images of the nanocomposites showed well-distributed nanofillers in the melted PLA matrix (Figure 11), which indicates that the dip coating method on nonwoven mats is an effective approach for obtaining a uniform and homogeneous nanofiller distribution in the polymer matrix. After cyclic loading, the extensively extruded sheet-like debris signifies the extensive deformation of GNP-filled PLA nanocomposite through the GNP network. The thermally reduced graphene oxide nanosheets form a compact and overlapping network at the original state of the nanocomposite specimen. With increased strain levels and cyclic loading, the deformation of the PLA polymer matrix transfers to the graphene sheets, leading to slippage of the neighbouring graphene sheets, so the GNPs become loose and the overlapping area decreases [16]. With further increase in strain level, the GNP may fracture. Once a crack is generated, it expands rapidly, and the resistance of graphene increases greatly and eventually becomes infinite. Moreover, the defects in the cotton fibre-reinforced PLA matrix composites also accelerate the deterioration of the sensing materials. 

## 4. Conclusions

This work successfully fabricated cotton/PLA composites via a scalable process and introduced homogeneously dispersed and reduced graphene nanoplatelets through simple dip coating on nonwoven mats followed by thermal consolidation. With 0.2 wt% GO addition, the nanohardness of the cured nanocomposite enhanced 30 times, and the elastic modulus enhanced 32 times in comparison with the pristine cotton/PLA composites. With such a small percentage of GNP inclusion, the cotton/PLA composites transformed from insulating to semi-conducting. The electrical resistivity measured by a four-point probe tester was 6.10 Ω∙cm for the composite with 0.4 wt% of GO and 5.88 Ω∙cm for that with 0.5 wt% of GO, respectively. Both the thermal conductivity and thermal diffusivity increased due to the deposition of thermally-reduced GO on the surface of the cotton/PLA composites. The strain sensing behaviour under quasi-static and dynamic tensile loading of nanocomposites was investigated. Up to a maximum strain of 1%, the strain-dependent resistance showed good recoverability and reproducibility after a few stabilisation cycles. The gauge factor of this biocomposite sensor is 2.59, which paves the way for potential use in structural health monitoring or other sensing purposes in environmentally friendly lightweight materials. 

The advantages of the dip coating method are its simplicity and economical aspects, which can meet modest standard requirements for a competitive cost. This approach is, however, more suited to producing graphene-based composite coatings than pristine graphene coatings due to the higher viscosity of the composite coating and stronger interfacial adhesion to the substrate. Graphene-based composite coatings can be more uniform and thicker than pristine graphene coatings for effectively introducing additional functionalities.

## Data Availability

The data presented in this study are available on request from the corresponding author. The data are not publicly available due to privacy.

## References

[1] Pickering K.L., Efendy M.G.A., Le T.M. (2016). A Review of Recent Developments in Natural Fibre Composites and Their Mechanical Performance. Compos. Part A.

[2] Gurunathan T., Mohanty S., Nayak S.K. (2015). A Review of the Recent Developments in Biocomposites Based on Natural Fibres and Their Application Perspectives. Compos. Part A.

[3] Awais H., Nawab Y., Amjad A., Anjang A., Md Akil H., Zainol Abidin M.S. (2021). Environmental Benign Natural Fibre Reinforced Thermoplastic Composites: A Review. Compos. Part C.

[4] Mochane M.J., Magagula S.I., Sefadi J.S., Mokhena T.C. (2021). A Review on Green Composites Based on Natural Fiber-Reinforced Polybutylene Succinate (Pbs). Polymers.

[5] Stark N.M., White R.H., Mueller S.A., Osswald T.A. (2010). Evaluation of Various Fire Retardants for Use in Wood Flour–Polyethylene Composites. Polym. Degrad. Stab..

[6] Azwa Z.N., Yousif B.F., Manalo A.C., Karunasena W. (2013). A Review on the Degradability of Polymeric Composites Based on Natural Fibres. Mater. Des..

[7] Asyraf M.R.M., Syamsir A., Zahari N.M., Supian A.B.M., Ishak M.R., Sapuan S.M., Sharma S., Rashedi A., Razman M.R., Zakaria S.Z.S. (2022). Product Development of Natural Fibre-Composites for Various Applications: Design for Sustainability. Polymers.

[8] Wang J., Bao L., Zhao H., Lei J. (2012). Preparation and Characterization of Permanently Anti-Static Packaging Composites Composed of High Impact Polystyrene and Ion-Conductive Polyamide Elastomer. Compos. Sci. Technol..

[9] Lukawski D., Hochmanska-Kaniewska P., Janiszewska D., Wroblewski G., Patmore J., Lekawa-Raus A. (2022). Enriching Wpcs and Nfpcs with Carbon Nanotubes and Graphene. Polymers.

[10] Tamburrano A., Sarasini F., De Bellis G., D’Aloia A.G., Sarto M.S. (2013). The Piezoresistive Effect in Graphene-Based Polymeric Composites. Nanotechnology.

[11] Zhang J., Wang J., Lin T., Wang C.H., Ghorbani K., Fang J., Wang X. (2014). Magnetic and Mechanical Properties of Polyvinyl Alcohol (Pva) Nanocomposites with Hybrid Nanofillers—Graphene Oxide Tethered with Magnetic Fe3o4 Nanoparticles. Chem. Eng. J..

[12] Mahmoud Zaghloul M.Y., Yousry Zaghloul M.M., Yousry Zaghloul M.M. (2021). Developments in Polyester Composite Materials—An in-Depth Review on Natural Fibres and Nano Fillers. Compos. Struct..

[13] Tian H., Shu Y., Wang X.F., Mohammad M.A., Bie Z., Xie Q.Y., Li C., Mi W.T., Yang Y., Ren T.L. (2015). A Graphene-Based Resistive Pressure Sensor with Record-High Sensitivity in a Wide Pressure Range. Sci. Rep..

[14] Qin Y., Peng Q., Ding Y., Lin Z., Wang C., Li Y., Xu F., Li J., Yuan Y., He X. (2015). Lightweight, Superelastic, and Mechanically Flexible Graphene Polyimide Nanocomposite Foam for Strain Sensor Application. ACS Nano.

[15] Chen Z., Ren W., Gao L., Liu B., Pei S., Cheng H.M. (2011). Three-Dimensional Flexible and Conductive Interconnected Graphene Networks Grown by Chemical Vapour Deposition. Nat. Mater..

[16] Wu S., Ladani R.B., Zhang J., Ghorbani K., Zhang X., Mouritz A.P., Kinloch A.J., Wang C.H. (2016). Strain Sensors with Adjustable Sensitivity by Tailoring the Microstructure of Graphene Aerogel/Pdms Nanocomposites. ACS Appl. Mater. Interfaces.

[17] Smith A.D., Niklaus F., Paussa A., Schroder S., Fischer A.C., Sterner M., Wagner S., Vaziri S., Forsberg F., Esseni D. (2016). Piezoresistive Properties of Suspended Graphene Membranes under Uniaxial and Biaxial Strain in Nanoelectromechanical Pressure Sensors. ACS Nano.

[18] Zhu S.E., Krishna Ghatkesar M., Zhang C., Janssen G.C.A.M. (2013). Graphene Based Piezoresistive Pressure Sensor. Appl. Phys. Lett..

[19] Deng C., Jiang J., Liu F., Fang L., Wang J., Li D., Wu J. (2015). Influence of Graphene Oxide Coatings on Carbon Fiber by Ultrasonically Assisted Electrophoretic Deposition on Its Composite Interfacial Property. Surf. Coat. Technol..

[20] Kuilla T., Bhadra S., Yao D., Kim N.H., Bose S., Lee J.H. (2010). Recent Advances in Graphene Based Polymer Composites. Prog. Polym. Sci..

[21] Du S.-S., Li F., Xiao H.-M., Li Y.-Q., Hu N., Fu S.-Y. (2016). Tensile and Flexural Properties of Graphene Oxide Coated-Short Glass Fiber Reinforced Polyethersulfone Composites. Compos. Part B.

[22] Mahmood H., Tripathi M., Pugno N., Pegoretti A. (2016). Enhancement of Interfacial Adhesion in Glass Fiber/Epoxy Composites by Electrophoretic Deposition of Graphene Oxide on Glass Fibers. Compos. Sci. Technol..

[23] Song N., Gao Z., Li X. (2020). Tailoring Nanocomposite Interfaces with Graphene to Achieve High Strength and Toughness. Sci. Adv..

[24] Ummartyotin S., Manuspiya H. (2015). A Critical Review on Cellulose: From Fundamental to an Approach on Sensor Technology. Renew. Sustain. Energy Rev..

[25] Saba N., Tahir P., Jawaid M. (2014). A Review on Potentiality of Nano Filler/Natural Fiber Filled Polymer Hybrid Composites. Polymers.

[26] Karim N., Sarker F., Afroj S., Zhang M., Potluri P., Novoselov K.S. (2021). Sustainable and Multifunctional Composites of Graphene-Based Natural Jute Fibers. Adv. Sustain. Syst..

[27] Alomayri T., Low I.M. (2013). Synthesis and Characterization of Mechanical Properties in Cotton Fiber-Reinforced Geopolymer Composites. J. Asian Ceram. Soc..

[28] Zonatti W.F., Guimarães B.M.G., Duleba W., Ramos J.B. (2015). Thermoset Composites Reinforced with Recycled Cotton Textile Residues. Text. Cloth. Sustain..

[29] Rukmini K., Ramaraj B., Shetty S.K., Taraiya A., Bandyopadhyay S. (2013). Development of Eco-Friendly Cotton Fabric Reinforced Polypropylene Composites: Mechanical, Thermal, and Morphological Properties. Adv. Polym. Technol..

[30] Faruk O., Bledzki A.K., Fink H.-P., Sain M. (2014). Progress Report on Natural Fiber Reinforced Composites. Macromol. Mater. Eng..

[31] Piekarska K., Sowinski P., Piorkowska E., Haque M.M.U., Pracella M. (2016). Structure and Properties of Hybrid Pla Nanocomposites with Inorganic Nanofillers and Cellulose Fibers. Compos. Part A.

[32] Olonisakin K., Fan M., Xin-Xiang Z., Ran L., Lin W., Zhang W., Wenbin Y. (2021). Key Improvements in Interfacial Adhesion and Dispersion of Fibers/Fillers in Polymer Matrix Composites; Focus on Pla Matrix Composites. Compos. Interfaces.

[33] Ali A., Andriyana A. (2020). Properties of Multifunctional Composite Materials Based on Nanomaterials: A Review. RSC Adv..

[34] George G., Dev A.P., Asok N.N., Anoop M.S., Anandhan S. (2021). Dispersion Analysis of Nanofillers and Its Relationship to the Properties of the Nanocomposites. Mater. Today Proc..

[35] Mohammed Basheer E.P., Marimuthu K. (2022). Carbon Fibre-graphene Composite Polylactic acid (PLA) Material for COVID Shield Frame. Mater. Und Werkst..

[36] Pierleoni D., Xia Z.Y., Christian M., Ligi S., Minelli M., Morandi V., Doghieri F., Palermo V. (2016). Graphene-based Coatings on Polymer Films for Gas Barrier Applications. Carbon.

[37] Rouhani S., Hosseinnezhad M., Sohrab N., Gharanjig K., Salem A., Ranjbar Z. (2022). Investigation of the Effect of rGO/TiO_2_ on Photovoltaic Performance of DSSCs Devices. Prog. Color Colorants Coat..

[38] Hummers W.S., Offeman R.E. (1958). Preparation of Graphitic Oxide. J. Am. Chem. Soc..

[39] Kongahge D., Foroughi J., Gambhir S., Spinks G.M., Wallace G.G. (2016). Fabrication of a Graphene Coated Nonwoven Textile for Industrial Applications. RSC Adv..

[40] Krishnamoorthy K., Navaneethaiyer U., Mohan R., Lee J., Kim S.-J. (2011). Graphene Oxide Nanostructures Modified Multifunctional Cotton Fabrics. Appl. Nanosci..

[41] Rabiei M., Raziyan M.S., Ebrahimi-Kahrizsangi R., Nasiri S., Palevicius A., Janusas G., Vilkauskas A. (2022). Effects of 5 wt.% Polycaprolactone, Polyhydroxybutyrate and Polyvinyltrimethoxysilane on the Properties of Ag/Zn/Mg Alloy. Polymers.

[42] Ren P.G., Yan D.X., Ji X., Chen T., Li Z.M. (2011). Temperature Dependence of Graphene Oxide Reduced by Hydrazine Hydrate. Nanotechnology.

[43] Gao W., Alemany L.B., Ci L., Ajayan P.M. (2009). New Insights into the Structure and Reduction of Graphite Oxide. Nat. Chem..

[44] Allen M.J., Tung V.C., Kaner R.B. (2010). Honeycomb Carbon a Review of Graphene. Chem. Rev..

[45] Lu J., Peng Q., Wang W., Nan C., Li L., Li Y. (2013). Nanoscale Coating of Limo_2_ (M = Ni, Co, Mn) Nanobelts with Li^+^-Conductive Li_2_TiO_3_: Toward Better Rate Capabilities for Li-Ion Batteries. J. Am. Chem. Soc..

[46] Burger N., Laachachi A., Ferriol M., Lutz M., Toniazzo V., Ruch D. (2016). Review of Thermal Conductivity in Composites: Mechanisms, Parameters and Theory. Prog. Polym. Sci..

[47] Venkanna B.K. (2010). Fundamentals of the Heat and Mass Transfer.

[48] Martin-Gallego M., Verdejo R., Khayet M., de Zarate J.M.O., Essalhi M., Lopez-Manchado M.A. (2011). Thermal Conductivity of Carbon Nanotubes and Graphene in Epoxy Nanofluids and Nanocomposites. Nanoscale Res. Lett..

[49] Wang D.Y., Tao L.Q., Liu Y., Zhang T.Y., Pang Y., Wang Q., Jiang S., Yang Y., Ren T.L. (2016). High Performance Flexible Strain Sensor Based on Self-Locked Overlapping Graphene Sheets. Nanoscale.

[50] Hu N., Karube Y., Yan C., Masuda Z., Fukunaga H. (2008). Tunneling Effect in a Polymer/Carbon Nanotube Nanocomposite Strain Sensor. Acta Mater..

[51] Hempel M., Nezich D., Kong J., Hofmann M. (2012). A Novel Class of Strain Gauges Based on Layered Percolative Films of 2d Materials. Nano Lett..

